# Searching for randomized controlled trials and systematic reviews on exercise. A descriptive study: Busca de ensaios clínicos randomizados e revisões sistemáticas sobre exercício físico. Um estudo descritivo

**DOI:** 10.1590/1516-3180.2013.8040011

**Published:** 2014-12-19

**Authors:** Antonio José Grande, Tammy Hoffmann, Paul Glasziou

**Affiliations:** I BSc, MSc. Master’s Student in the Postgraduate Program on Internal Medicine and Therapeutics, Federal University of São Paulo (Escola Paulista de Medicina, Universidade Federal de São Paulo, EPM-Unifesp), São Paulo, Brazil.; II PhD. Centre for Research in Evidence-Based Practice, Faculty of Health Sciences and Medicine, Bond University, Gold Coast, Queensland, Australia.

**Keywords:** Motor activity, Health information management, Health communication, Research, Biomedical research

## Abstract

**CONTEXT AND OBJECTIVE:**

The current paradigm of science is to accumulate as much research data as possible, with less thought given to navigation or synthesis of the resulting mass, which hampers locating and using the research. The aim here was to describe the number of randomized controlled trials (RCTs) and systematic reviews (SRs) focusing on exercise, and their journal sources, that have been indexed in PubMed over time.

**DESIGN AND SETTING:**

Descriptive study conducted at Bond University, Australia.

**METHOD:**

To find RCTs, a search was conducted in PubMed Clinical Queries, using the category “Therapy” and the Medical Subject Headings (MeSH) term “Exercise”. To find SRs, a search was conducted in PubMed Clinical Queries, using the category “Therapy”, the MeSH term “Exercise” and various methodological filters.

**RESULTS:**

Up until 2011, 9,354 RCTs about exercise were published in 1,250 journals and 1,262 SRs in 513 journals. Journals in the area of Sports Science published the greatest number of RCTs and journals categorized as belonging to “Other health professions” area (for example nursing or psychology) published the greatest number of SRs. The Cochrane Database of Systematic Reviews was the principal source for SRs, with 9.8% of the total, while the Journal of Strength and Conditioning Research and Medicine & Science in Sports & Exercise published 4.4% and 5.0% of the RCTs, respectively.

**CONCLUSIONS:**

The rapid growth and resulting scatter of RCTs and SRs on exercise presents challenges for locating and using this research. Solutions for this issue need to be considered.

## RESUMO

**CONTEXTO E OBJETIVO:**

O paradigma atual da ciência é acumular o máximo de dados de pesquisa possível, com menos atenção dada a navegação ou a síntese do volume resultante, o que dificulta a localização e utilização das pesquisas. O objetivo foi descrever o número de ensaios clínicos randomizados (ECRs) e revisões sistemáticas (RSs) focados em exercício físico publicados em periódicos, que foram indexados no PubMed ao longo do tempo.

**TIPO DE ESTUDO E LOCAL:**

Estudo descritivo conduzido na Bond University, Austrália.

**MÉTODO:**

Para encontrar ECRs, foi realizada uma busca no PubMed Clinical Queries, usando a categoria “Terapia” e o termo de Medical Subject Headings (MeSH) “Exercício”. Para encontrar RSs, foi realizada uma busca no PubMed Clinical Queries, usando a categoria “Therapy” e o termo MeSH “Exercício” e vários filtros.

**RESULTADOS:**

Até 2011, 9.354 ECRs sobre exercício foram publicados em 1.250 periódicos e 1.262 RSs em 513 periódicos. Periódicos na área de Ciências do Esporte publicaram o maior número de ECRs e revistas classificadas como pertencentes à area “Outras profissões de saúde” (por exemplo, enfermagem ou psicologia) publicaram o maior número de RSs. A Cochrane Database of Systematic Reviews foi a principal fonte para RSs, com 9,8% do total, enquanto o Journal of Strength and Conditioning Research e o Medicine & Science Sports & Exercise publicaram 4,4% e 5,0% dos ECRs, respectivamente.

**CONCLUSÃO:**

O rápido crescimento e consequente dispersão dos ECRs e RSs sobre exercício físico apresentam desafios para localizar e usar pesquisas. Precisa-se de reflexões sobre a resolução do assunto.

## INTRODUCTION

The current paradigm of science is to accumulate as much research data as possible,^1^ with less thought given to navigation or synthesis of the resulting mass. Research output, in the form of journal articles, is doubling about every seven years.^2^ This research tsunami has generated problems for users, making finding, reading and applying this information in decision-making increasingly problematic. In 1994, it was estimated that a professional had to read 17 to 20 original papers every day to keep up to date,^2^ and this figure is estimated to have increased 8-fold since then. Over time, the research community has become more critical of the quality of the information and there have been, and continue to be, many efforts to create methods to sift, evaluate and synthesize what has been published.^3^

To answer questions about the effectiveness of interventions, the study designs that provide the highest levels of evidence are randomized controlled trials (RCTs), as primary research, and systematic reviews (SRs) for research synthesis.^4^^,^^5^ These two methods are important for decision-making and the number of published papers that have used these study designs is extremely high and increasing rapidly: as of May 2012, over 2.3 million RCTs and 127,000 SRs were indexed in Medline.^6^

Exercise, for both sports and medical conditions, has been the focus of many RCTs and SRs.^7^^,^^8^^,^^9^^,^^10^ Due to its broad applicability, exercise is used for health promotion and public health interventions, for treating disease and for preventive measures.^7^^,^^8^^,^^9^ Its importance on a daily basis is highlighted in many studies.^9^^,^^10^^,^^11^ However, it is becoming a growing challenge for professionals to be aware of this research and to be able to find it. The “scatter” of research has been studied in a number of specialties, but not in exercise.^12^ The aim of our study was to describe the number of RCTs and SRs focusing on exercise, and their journal sources, which have been indexed in Medline over time.

## METHODS

### Selection of studies

This was a descriptive study conducted at Bond University, Australia, during the months of January and February 2013.

An initial pilot search, conducted independently by AJG and PG, tested the specificity and sensitivity of using the term “Exercise” with the Therapy/narrow filter in PubMed Clinical Queries. This returned 75,367 results; we checked the 100 results from PubMed, in both the Trials and the Systematic Reviews columns, to assess whether the studies returned in the search matched the topic. Only approximately 40% were RCTs or SRs that had evaluated exercise. It is worth noting that searching for the Medical Subject Heading (MeSH) term “Exercise” also allows synonyms such as: “(Exercises); (Exercise, Physical); (Exercises, Physical); (Physical Exercise); (Physical Exercises); (Exercise, Isometric); (Exercises, Isometric); (Isometric Exercises); (Isometric Exercise); (Exercise, Aerobic); (Aerobic Exercises); (Exercises, Aerobic); (Aerobic Exercise)”.

A modified search, with consensus among the authors, was conducted only by AJG in PubMed to find all RCTs involving exercise, and consisted of the MeSH term “Exercise [MeSH]” in PubMed Clinical Queries, selecting the category “Therapy” and the scope “Narrow”, and limited to “Humans”. To find SRs, the following strategy was used: (systematic [tiab] AND review [tiab]) OR meta-analysis [pt] OR CDSR [so] AND Exercise [MeSH], in PubMed Clinical Queries, with the category “Therapy” and the scope “Narrow” selected.

The search was carried out by AJG in PubMed on January 22, 2013. We included RCTs and SRs that had been published from the beginning of indexed records until the end of 2011. We excluded those published in 2012, since indexation of these articles was incomplete at the time of searching.

### Data analysis

The RCTs and SRs were stratified by year and by journal. We considered the broad category of journal type that they were published in, using the following five categories: Sport Sciences; Rehabilitation; Medicine (general and internal); Medical Specialty; and other areas (for example nutrition or nursing). The classification of journals followed the categories used by ISI Web of Knowledge.

All references were exported from PubMed to Endnote X6, and then an output style was developed and exported to Excel 2010. All records were individually classified by AJG in a computer spreadsheet. Data were analyzed descriptively.

## RESULTS

We found that 9,354 RCTs about exercise had been published in 1,250 journals up to the end of 2011, and 1,262 SRs in 513 journals. Table 1 presents the numbers of RCTs and SRs per journal category.

**Table 1.**
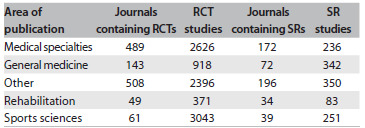
Number of randomized controlled trials (RCTs) and systematic reviews (SRs) on exercise per journal category

**Figure 1.**
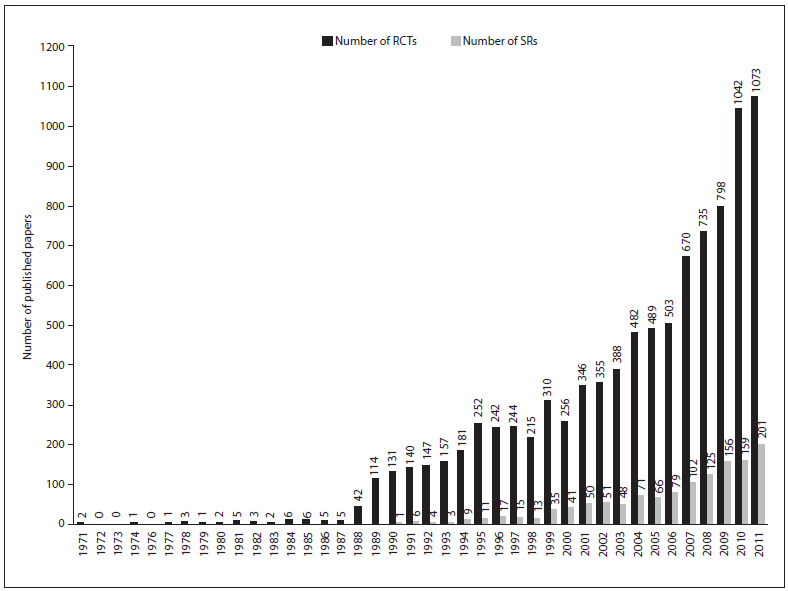
Number of randomized controlled trials (RCTs) and systematic reviews (SRs) on exercise published each year.

Figure 1 shows the numbers of RCTs and SRs published each year. The first RCT about exercise was published in 1971 and the first SR was published 20 years later, in 1991. After 1988, the number of RCTs published increased dramatically, whereas the number of SRs published per year only exceeded 100 in 2007.

**Figure 2.**
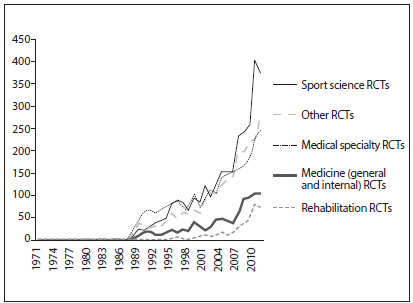
Number of randomized controlled trials (RCTs) on exercise published each year, in each broad area of knowledge.

Figure 2 shows the number of RCTs published each year, across each of the areas. Two areas (Others and Medical Specialties) had similar growth trends regarding the numbers of published papers, while Sport Sciences had sharper growth. The areas of Medicine (General and Internal) and Rehabilitation showed less growth.

**Figure 3.**
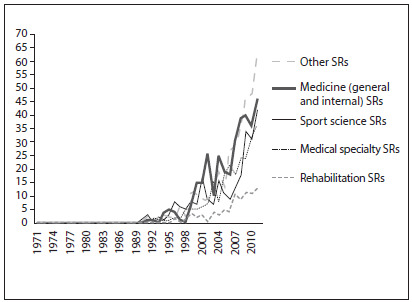
Number of systematic reviews (SRs) on exercise published each year, in each broad area of knowledge.

Figure 3 shows the number of SRs published each year, across each of the areas. In contrast to the growth in RCTs, the growth trend for the number of SRs published was similar in four of the five areas, with only the Rehabilitation area showing slower growth in the number of published SRs.

**Figure 4.**
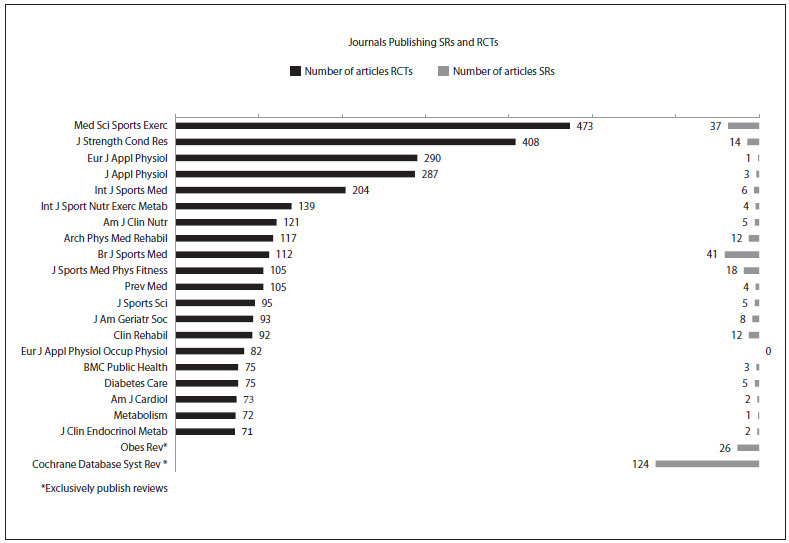
The 20 journals that have published the highest numbers of randomized controlled trials (RCTs) on exercise, as well as the number of systematic reviews (SRs) on exercise that these journals have published.

Figure 4 shows the top 20 journals, which have published the highest numbers of RCTs. It also shows the corresponding numbers of SRs that have been published by these journals. The journal that has published the highest number of systematic reviews about exercise is the Cochrane Database of Systematic Reviews (n = 124), followed by the British Journal of Sports Medicine (n = 41).

## DISCUSSION

Exercise is a well-known intervention, which historically has been studied using an observational approach.^9^ One limitation with observational research is that the cause-effect relationship between variables cannot be considered strong.^12^ Additionally, there are many confounding variables that can lead to misinterpretation in observational studies. RCTs and SRs provide stronger evidence for the effectiveness of exercise as an intervention.^13^^,^^14^^,^^15^ However, the exponential growth in RCTs and SRs that aim to provide higher quality of evidence about the effectiveness of interventions is occurring in many areas of knowledge,^2^^,^^6^^,^^16^ thus leading to risks relating to this exponential growth. These risks need further discussion within the academic sphere.^4^^,^^12^

Since exercise is relevant to many disciplines and it is a growing concept for health promotion and rehabilitation, it is important to reflect about its types (yoga, Pilates and tai ji, among others) and where their MeSH trees are best included. One piece of advice in this regard would be to recall the definition of exercise and physical activity. The classical definition of exercise is that it is *“a subset of physical activity that is planned, structured and repetitive and has as a final or an intermediate objective the improvement or maintenance of physical fitness*” and the definition of physical activity is “*any bodily movement produced by skeletal muscles that results in energy expenditure*”.^17^ Since many more terms are likely to emerge, their scatter becomes more of a problem and finding relevant information becomes more difficult.

In our study, we found that over 1100 journals are publishing RCTs and over 440 journals are publishing SRs about exercise. These data shows that it is almost impossible to keep up to date in the traditional “push” manner; using “just-in-case” information, where information is “pushed” to the user.^12^^,^^18^^,^^19^ For example, if a user subscribed to the top ten journals (or even just their table of contents) in the year 2011, that would represent being made aware of 26% of the total number of published RCTs. A recent study highlighted the enormous problem of the scatter of RCTs and SRs across numerous medical specialties and subspecialties, with the problem much more expansive in some disciplines (such as neurology) than in others.^12^ Our results showed that there is variation in the volume of RCTs and SRs about exercise that journals in various broad knowledge areas publish.

This study has some limitations. Only one database (PubMed) was used for our search, although this is the largest database containing biomedical and health publications. It is possible that some of the studies that were included were not relevant to exercise, since we did not read the full text of each article. Conversely, it is also possible that some RCTs and SRs relevant to exercise were missed by our search.

Although RCTs and SRs provide the best study designs for evaluating intervention effectiveness, users of exercise data need skills in a) locating exercise research and b) appraising it for its quality, either by having skills in critical appraisal or by using some of the existing solutions that exist for providing filtered, pre-appraised research (e.g. PEDro database or McMaster PLUS). We conducted a quick search on August 25, 2013, using “Exercise” in McMaster PLUS and in PEDro, and found 2,097 and 8,116 pre-appraised studies, respectively.

Exercise is relevant to so many disciplines and such broad knowledge areas that methods for providing easier access to high quality exercise research are needed. We also believe that is time to discuss not only besides methodology but also the description of exercise programs, in order to improve their practical application.^19^^,^^20^^,^^21^^,^^22^^,^^23^

## CONCLUSION

There has been rapid growth in the number of RCTs and SRs relevant to exercise over the last 15-20 years. Exercise RCTs and SRs have been published in journals in the Sports Science area more than in any other area (31% of the total). We found that one particular journal (Cochrane Database of Systematic Reviews) is the principal source for exercise SRs; and two journals (Journal of Strength and Conditioning Research and Medicine & Science in Sports & Exercise) published the highest number of exercise RCTs. This growth and scatter of exercise RCTs and SRs presents challenges for locating and using this research, and solutions for this issue need to be considered.
